# Approaches to estimate bidirectional causal effects using Mendelian randomization with application to body mass index and fasting glucose

**DOI:** 10.1371/journal.pone.0293510

**Published:** 2024-03-08

**Authors:** Jinhao Zou, Rajesh Talluri, Sanjay Shete

**Affiliations:** 1 Department of Biostatistics, The University of Texas MD Anderson Cancer Center, Houston, Texas, United States of America; 2 Department of Data Science, The University of Mississippi Medical Center, Jackson, Mississippi, United States of America; 3 Department of Epidemiology, The University of Texas MD Anderson Cancer Center Houston, Texas, United States of America; University of Turin: Universita degli Studi di Torino, ITALY

## Abstract

Mendelian randomization (MR) is an epidemiological framework using genetic variants as instrumental variables (IVs) to examine the causal effect of exposures on outcomes. Statistical methods based on unidirectional MR (UMR) are widely used to estimate the causal effects of exposures on outcomes in observational studies. To estimate the bidirectional causal effects between two phenotypes, investigators have naively applied UMR methods separately in each direction. However, bidirectional causal effects between two phenotypes create a feedback loop that biases the estimation when UMR methods are naively applied. To overcome this limitation, we proposed two novel approaches to estimate bidirectional causal effects using MR: BiRatio and BiLIML, which are extensions of the standard ratio, and limited information maximum likelihood (LIML) methods, respectively. We compared the performance of the two proposed methods with the naive application of UMR methods through extensive simulations of several scenarios involving varying numbers of strong and weak IVs. Our simulation results showed that when multiple strong IVs are used, the proposed methods provided accurate bidirectional causal effect estimation in terms of median absolute bias and relative median absolute bias. Furthermore, compared to the BiRatio method, the BiLIML method provided a more accurate estimation of causal effects when weak IVs were used. Therefore, based on our simulations, we concluded that the BiLIML should be used for bidirectional causal effect estimation. We applied the proposed methods to investigate the potential bidirectional relationship between obesity and diabetes using the data from the Multi-Ethnic Study of Atherosclerosis cohort. We used body mass index (BMI) and fasting glucose (FG) as measures of obesity and type 2 diabetes, respectively. Our results from the BiLIML method revealed the bidirectional causal relationship between BMI and FG in across all racial populations. Specifically, in the White/Caucasian population, a 1 kg/m^2^ increase in BMI increased FG by 0.70 mg/dL (95% confidence interval [CI]: 0.3517–1.0489; *p* = 8.43×10^−5^), and 1 mg/dL increase in FG increased BMI by 0.10 kg/m^2^ (95% CI: 0.0441–0.1640; *p* = 6.79×10^−4^). Our study provides novel findings and quantifies the effect sizes of the bidirectional causal relationship between BMI and FG. However, further studies are needed to understand the biological and functional mechanisms underlying the bidirectional pathway.

## Introduction

Causal inference is of vital importance in several fields of medicine and epidemiology [1,2]. It is used to identify factors causally associated with common diseases, thereby providing a basis for disease intervention and prevention [2,3]. Randomized controlled trials (RCTs) have been used for measuring the causal effects of treatments on outcomes [4–6]. However, RCTs may be expensive, with longer follow-up and potentially multiple ethical problems in real-life applications [2,4–6]. Alternately, an observational study design is commonly used to identify an association between treatment and outcome [7]. In traditional prospective observational cohort studies, exposure is measured, and then participants are followed over time to find out how many develop certain health conditions. In retrospective cohort studies, subjects are selected based on preexisting exposure status, and outcome data from the past are assembled for analysis. In case-control studies, subjects are selected and categorized into the case or control group based on the incidence of outcomes. The exposures are measured retrospectively for both the case and control groups for analysis [7,8]. Inferences from observational studies can be biased by unobserved confounders that affect both the exposure and outcome or by potential reverse causations [4,6,9–11]. For example, an observational study identified an association between coronary heart disease and vitamin E intake, but an RCT found no such association [12,13].

Methods using instrumental variables (IVs) were proposed as an alternative solution to examine the causality between exposure and outcome using cross-sectional observational datasets. An IV is a factor that is predictive of exposure but is not directly associated with either the outcome or confounders [14,15]. Mendel’s laws of inheritance state that alleles are randomly distributed from parents to offspring, so an allele-related trait also separates randomly in a population, and those alleles are unlikely to be associated with confounders [6,10]. Also, the germline genotypes are assigned to individuals before any possible exposures and outcomes, thus reducing the concern of reverse causation [6,10]. Therefore, causal inference methods proposed using genetic variants as IVs can reduce bias due to unobserved confounders and reverse causation [6]. Mendelian randomization (MR) is a framework used to infer the causal relationship between exposure and outcome using genetic variants as IVs of exposure of interest [4,6,11,15,16].

Correctly selecting IVs is critical to a successful MR study. There are three assumptions of selecting valid IVs in MR studies: 1) the genetic variables are associated with the exposure, 2) the genetic variables are independent of confounders, and 3) the genetic variables are independent of the outcome given the exposures and all confounders [4,6,9,17]. During recent decades, thousands of genetic associations have been revealed by genome-wide association studies [2,6,18], which provide a reliable source of candidate IVs for MR studies. Several MR methods have been proposed on the foundations of the three assumptions listed above [2,19–21].

A commonly used MR method is the ratio method with an IV, which uses the ratio of the coefficient of regressing outcome on an IV and the coefficient of regressing exposure on the IV as the estimate of the causal effect between exposure and outcome [2,19,21]. This method has been expanded to multiple IVs using inverse-variance weighted (IVW) methods. With uncorrelated IVs, the IVW estimator from MR studies combines the ratio estimates from each IV through IVW meta-analysis [19,20].

One challenge of performing MR studies is that many genetic variants are only modestly associated with the exposure and explain only a small amount of the exposure’s variance [22]. The F-statistic of regressing the exposure on a genetic variant is commonly used in MR studies to measure the strength of the genetic variant as an IV. When a genetic variant is an IV for exposure, and the associated F-statistic is less than 10, it is considered a weak IV [23,24]. Finding strong IVs for MR studies is often difficult. When multiple weak IVs are used, estimations from the IVW ratio method will be biased [20,25]. For instance, studies with weak IVs can be sensitive to violations of the IV assumptions, leading to biased effect estimates [15,26]. To overcome bias associated with weak IVs, limited information maximum likelihood (LIML) estimators have been proposed. Theoretical justifications and simulation studies have shown that the LIML estimators provide accurate estimation even when weak IVs are used [22,27].

**Fig 1:**
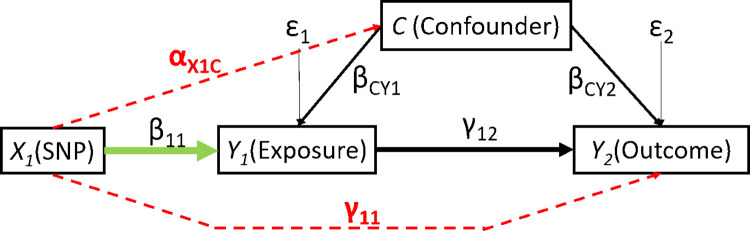
Unidirectional Mendelian randomization model.

**Fig 2:**
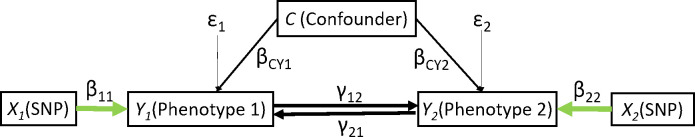
Bidirectional Mendelian randomization model with a feedback loop.

Although the ratio and LIML MR methods can estimate unidirectional causal effects (Fig 1), many phenotypes have bidirectional causal effects (Fig 2) in which the exposure and the outcome affect each other, such as the bidirectional relationship between diabetes and obesity [28], between inflammation and sleep disorders [29], or between depression and pain [30]. The bidirectional relationship between exposure and outcome leads to a feedback loop. Typically, bidirectional causal effects are estimated using two unidirectional MR (UMR) models, one for each causal direction [31,32]. When the bidirectional causal effects are estimated using UMR methods for each direction separately, the feedback loop will bias the estimation of causal effects [10,17]. Darrous et al. have proposed a method for estimating bidirectional causal effects based on summary data; however, their model applies to two-sample MR [33]. Although several MR-related reviews addressed the existence of a feedback loop in bidirectional causation scenarios [5,10,17], to our knowledge, no MR method for the estimation of bidirectional causal effects accounts for the feedback loop.

In this manuscript, we propose two methods to estimate bidirectional causal effects and account for the feedback loop between exposure and outcome in a one-sample bidirectional MR (BMR) model. The proposed BiRatio method and BiLIML method are extended from the traditional ratio and LIML methods, respectively. We compared the performance via simulations when the underlying model is unidirectional and bidirectional with different strengths of IVs and found that the BiRatio and BiLIML methods provide an accurate estimation of causal effects. We applied the proposed methods to estimate the effects of the bidirectional relationship between obesity and diabetes: Observational studies and RCTs have shown that individuals with higher body mass index (BMI) also have a higher likelihood of developing diabetes [28,34,35], and patients with type 2 diabetes have a higher likelihood of being obese [28]. With BMI as a measure of obesity and fasting glucose (FG) as a measure of diabetes, we investigated the bidirectional relationship between BMI and FG by estimating causal effects using data from the Multi-Ethnic Study of Atherosclerosis (MESA) cohort.

## Methods

The study is approved by MD Anderson institutional review board and uses secondary data from the dbGaP.

### Unidirectional MR model

The UMR model is shown in Fig 1. Let Y_1_ denote exposure of interest and Y_2_ denote the outcome of interest. Let X_1_ denote a SNP (or a set of SNPs) that is only associated with Y_1_ but is not directly associated with Y_2_. Let C represent the (typically unmeasured) confounder that affects both the exposure and outcome. The model in Fig 1 can be represented by the following sets of equations:

$$Y_{1}=\beta_{01}+\beta_{11}X_{1}+\beta_{CY1}C+\varepsilon_{1}$$ (1)

$$Y_{2}=\beta_{02}+\gamma_{12}Y_{1}+\beta_{CY2}C+\varepsilon_{2}$$ (2)

### Bidirectional MR model

The BMR model is shown in Fig 2. Let Y_1_ and Y_2_ denote outcomes of interest where each outcome is causally related to the other. Let X_1_ denote a SNP that is only associated with Y_1_ but not directly associated with Y_2_. Similarly, let X_2_ denote a SNP that is only associated with only Y_2_ but not directly associated with Y_1_. The bidirectional model is represented by a joint system of equations:

$$Y_{1}=\beta_{01}+\beta_{11}X_{1}+\beta_{CY1}C+\gamma_{21}Y_{2}+\varepsilon_{1}$$ (3)

$$Y_{2}=\beta_{02}+\beta_{22}X_{2}+\beta_{CY2}C+\gamma_{12}Y_{1}+\varepsilon_{2}$$ (4)

In this model, the bidirectional relationship between Y_1_ and Y_2_ leads to a recursive relationship (i.e., a feedback loop) between these two outcomes. After each feedback cycle, values of outcome variables Y_1_ and Y_2_ are altered. The feedback cycle converges to

$$Y_{1}=\frac{\beta_{01}+\gamma_{21}\beta_{02}}{1-\gamma_{12}\gamma_{21}}+\frac{\gamma_{21}\beta_{CY2}+\beta_{CY1}}{1-\gamma_{12}\gamma_{21}}C+\frac{\beta_{11}}{1-\gamma_{12}\gamma_{21}}X_{1}+\frac{\gamma_{21}\beta_{11}}{1-\gamma_{12}\gamma_{21}}X_{2}+\delta_{1}$$ (5)

$$Y_{2}=\frac{\beta_{02}+\gamma_{12}\beta_{01}}{1-\gamma_{12}\gamma_{21}}+\frac{\gamma_{12}\beta_{CY1}+\beta_{CY2}}{1-\gamma_{12}\gamma_{21}}C+\frac{\gamma_{12}\beta_{11}}{1-\gamma_{12}\gamma_{21}}X_{1}+\frac{\beta_{22}}{1-\gamma_{12}\gamma_{21}}X_{2}+\delta_{2}$$ (6)

when |*γ*_12_*γ*_21_|<1. See the S1 Appendix for derivation.

### Estimation methods

Various methods for the estimation of causal effects have been proposed [2,4,19,20]. One of the simplest MR methods is the ratio method [4,15]. For cases in which multiple IVs are used, the inverse-variance weighted (IVW) ratio method has been proposed [19,20].

### Unidirectional ratio method (Ratio)

The ratio estimator of γ_12_ is calculated using Eqs (1) and (2) as the ratio of coefficient of regression of Y_2_ on X_1_ and the coefficient of regression of Y_1_ on X_1_ [2,15].


$${\hat{\gamma }}_{12}=\frac{\hat{\gamma_{12}\beta_{11}}}{\hat{\beta_{11}}}$$ (7)

### Bidirectional ratio method (BiRatio)

In the literature [31,36–38], the ratio and IVW methods have been naively applied to Eqs (3) and (4) without accounting for the feedback cycle, leading to biased estimation. One study [31] used two UMR estimations for the causal effects in each direction. In our proposed approach, a joint system of Eqs (5) and (6) is used for parameter estimations. Specifically, γ_12_ is estimated as the ratio of the coefficient of regression of Y_2_ on X_1_ which is $\frac{\hat{\gamma_{12}\beta_{11}}}{1-\gamma_{12}\gamma_{21}}$ and the coefficient of regression of Y_1_ on X_1,_ which is $\frac{\hat{\beta_{11}}}{1-\gamma_{12}\gamma_{21}}$.


$$\hat{\gamma_{12}}=\frac{\hat{\frac{\gamma_{12}\beta_{11}}{1-\gamma_{12}\gamma_{21}}}}{\frac{\hat{\beta_{11}}}{1-\gamma_{12}\gamma_{21}}}$$ (8)

Although the bidirectional effect estimator in Eq (8) may look similar to the unidirectional MR ratio estimator in Eq (7), they are not equivalent because the estimated numerator and denominator include (1−*γ*_12_*γ*_21_) for the bidirectional method. Furthermore, the equations used to estimate γ_12_ also include X_2_ in Eq (4), which is not included in Eq (2) of the unidirectional model.

Similarly, γ_21_ is estimated as the ratio of the coefficient of regression of Y_1_ on X_2_ and the coefficient of regression of Y_2_ on X_2_.


$$\hat{\gamma_{21}}=\frac{\frac{\hat{\gamma_{21}\beta_{22}}}{1-\gamma_{12}\gamma_{21}}}{\frac{\hat{\beta_{22}}}{1-\gamma_{12}\gamma_{21}}}$$

When multiple IVs are used for estimating γ_12_, denoted by X_1.1_, …, X_1.k_, the IVW ratio estimator of γ_12_ is $\hat{\gamma_{12}}=\frac{{\sum {\left(\frac{\hat{\gamma_{12}\beta_{11}}}{1-\gamma_{12}\gamma_{21}}\right)}_{i}{\left(\frac{\hat{\beta_{11}}}{1-\gamma_{12}\gamma_{21}}\right)}_{i}\mathrm{var}{\left(\hat{\frac{\gamma_{12}\beta_{11}}{1-\gamma_{12}\gamma_{21}}}\right)}_{i})}^{-1}}{{\sum {\left(\frac{\hat{\beta_{11}}}{1-\gamma_{12}\gamma_{21}}\right)}_{i}^{2}\mathrm{var}({\left(\frac{\hat{\gamma_{12}\beta_{11}}}{1-\gamma_{12}\gamma_{21}}\right)}_{i})}^{-1}}$ where the ${\left(\hat{\frac{\gamma_{12}\beta_{11}}{1-\gamma_{12}\gamma_{21}}}\right)}_{i}$ is the coefficient of regression of Y_2_ on the X_1.i_, ${\left(\hat{\frac{\beta_{11}}{1-\gamma_{12}\gamma_{21}}}\right)}_{i}$ is the coefficient of regression of Y_1_ on X_1.i_, and the $\mathrm{var}({\left(\hat{\frac{\gamma_{12}\beta_{11}}{1-\gamma_{12}\gamma_{21}}}\right)}_{i})$ is the variance of coefficient from regression of Y_2_ on the X_1.i_, i = 1,…,k. When multiple IVs are used for estimating γ_21_, denoted by X_2.1_, …, X_2.k,_ the IVW ratio estimator of γ_21_ is $\hat{\gamma_{21}}=\frac{{\sum {\left(\frac{\hat{\gamma_{21}\beta_{22}}}{1-\gamma_{12}\gamma_{21}}\right)}_{i}{\left(\frac{\hat{\beta_{22}}}{1-\gamma_{12}\gamma_{21}}\right)}_{i}\mathrm{var}{\left(\hat{\frac{\gamma_{21}\beta_{22}}{1-\gamma_{12}\gamma_{21}}}\right)}_{i})}^{-1}}{{\sum {\left(\frac{\hat{\beta_{22}}}{1-\gamma_{12}\gamma_{21}}\right)}_{i}^{2}\mathrm{var}({\left(\frac{\hat{\gamma_{21}\beta_{22}}}{1-\gamma_{12}\gamma_{21}}\right)}_{i})}^{-1}}$ where the ${\left(\hat{\frac{\gamma_{21}\beta_{22}}{1-\gamma_{12}\gamma_{21}}}\right)}_{i}$ is the coefficient of regression of Y_1_ on the X_2.i_ ${\left(\hat{\frac{\beta_{22}}{1-\gamma_{12}\gamma_{21}}}\right)}_{i}$ is the coefficient of regression of Y_2_ on X_2.i_, and $\mathrm{var}({\left(\hat{\frac{\gamma_{21}\beta_{22}}{1-\gamma_{12}\gamma_{21}}}\right)}_{i})$ is the variance of coefficient from regression of Y_2_ on the X_2.i_, i = 1,…,k.

### Bidirectional LIML method (BiLIML)

As mentioned in the Introduction, many genetic variants are only modestly associated with the exposure and only explain a small amount of variance of the exposure [22]. When multiple weak IVs are used in MR studies, estimations from the IVW method will be biased [20,25]. However, the LIML method does not suffer from such bias, according to previous theoretical and simulation studies [22,27]. LIML was originally developed as an extension of the full information maximum likelihood (FIML) method. FIML estimates the parameters of simultaneous linear equation models using information from all equations. When one or more equations are mis-specified, FIML provides inconsistent estimations. LIML overcomes this disadvantage by using only information regarding the equation’s structure that includes the parameters of interest, such as the γ_12_ in Eq (2) from the UMR model for unidirectional causal effect estimation. LIML provides closed-form maximum likelihood estimates of the parameters (e.g., γ_12_) [15,39]. The LIML method was previously applied [32] using Eqs (1) and (2) to estimate the bidirectional causal effects between BMI and C-reactive protein; however, as mentioned above, such formulation ignores the feedback loop, leading to biased estimation. In our proposed approach, we adapt the LIML method to the BMR model using Eqs (5) and (6).

In our bidirectional LIML approach, we estimate γ_12_, using Eqs (4) and (5). We can rewrite Eq (5) as

$$Y_{1}=\alpha_{01}+\alpha_{CY1}C+\alpha_{11}X_{1}+\gamma_{23}X_{2}+\delta_{1}$$ (9)

where $\alpha_{01}=\frac{\beta_{01}+\gamma_{21}\beta_{22}}{1-\gamma_{12}\gamma_{21}},\;\alpha_{CY1}=\frac{\beta_{CY1}+\gamma_{21}\beta_{CY2}}{1-\gamma_{12}\gamma_{21}},\;\alpha_{11}=\frac{\beta_{11}}{1-\gamma_{12}\gamma_{21}}$, and $\gamma_{23}=\frac{\gamma_{21}\beta_{22}}{1-\gamma_{12}\gamma_{21}}$. Formulas (4) and (9) can be written as

$$\left[\begin{array}{cc} 1 & 0\\ -\gamma_{12} & 1 \end{array}\right]\left[\begin{array}{c} Y_{1}\\ Y_{2} \end{array}\right]=[\begin{array}{cccc} \alpha_{01} & \alpha_{CY1} & \alpha_{11} & \gamma_{23}\\ \beta_{02} & \beta_{CY2} & 0 & \beta_{22} \end{array}]\left[\begin{array}{c} 1\\ C\\ X_{1}\\ X_{2} \end{array}\right]+[\begin{array}{c} \delta_{1}\\ \varepsilon_{2} \end{array}]$$

which can be represented as $\Gamma Y=BW+{\left[\begin{array}{cc} \delta_{1} & \varepsilon_{2} \end{array}\right]}^{T}$, where $Y={\left[Y_{1}\;Y_{2}\right]}^{T},\Gamma =\left[\begin{array}{cc} 1 & 0\\ -\gamma_{12} & 1 \end{array}\right],W={\left[\begin{array}{cccc} 1 & C & X_{2} & X_{1} \end{array}\right]}^{T}$, and $B=[\begin{array}{cccc} \alpha_{01} & \alpha_{CY1} & \alpha_{11} & \gamma_{23}\\ \beta_{02} & \beta_{CY2} & 0 & \beta_{22} \end{array}]$. We assume $\left[\begin{array}{cc} \delta_{1} & \varepsilon_{2} \end{array}\right]$ follow multi-normal distribution. The likelihood function is

$$L={\left(2\pi \right)}^{n}\exp\left(-\frac{1}{2}{\left(\Gamma Y-BW\right)}^{T}(\Sigma^{-1}⨂I_{n}){\left(Y\Gamma -WB\right)}^{T}\right){\left|\Sigma ⨂I_{n}\right|}^{-\frac{1}{2}}$$ (10)

The maximum likelihood estimation of γ_12_ can be represented as

$${\left[\hat{\beta_{02}}\hat{\beta_{CY2}}\hat{\gamma_{12}}\right]}^{T}={\left(X^{T}(I_{n}-\hat{k}M_{w})X\right)}^{-1}X^{T}(I_{n}-\hat{k}M_{w})Y_{2}$$ (11)

where $X=[\begin{array}{ccc} 1 & \begin{array}{cc} C & X_{2} \end{array} & Y_{1} \end{array}],M_{w}=I_{n}-W{\left(W^{T}W\right)}^{-1}W^{T},W=[\begin{array}{ccc} 1 & \begin{array}{cc} C & X_{2} \end{array} & X_{1} \end{array}]$ and $\hat{k}$ is an eigenvalue of the matrix ${\left(Y^{T}M_{w}Y\right)}^{-1/2}Y^{T}M_{c}Y{\left(Y^{T}M_{w}Y\right)}^{-1/2}$, where *Y* = [*Y*_1_*Y*_2_], $M_{C}=I_{n}-C^{*}{\left({C^{*}}^{T}C^{*}\right)}^{-1}{C^{*}}^{T}$, and $C^{*}=[\begin{array}{cc} 1 & \begin{array}{cc} C & X_{2} \end{array} \end{array}]$.

Again, the estimated γ_12_ is different from the unidirectional LIML estimate because the estimation approach includes the instrumental variable, X_2_.

Similarly, in the bidirectional LIML approach, we can estimate γ_21_ using Eqs (3) and (6). We rewrite Eq (6) as

$$Y_{2}=\alpha_{02}+\alpha_{CY2}C+\alpha_{22}X_{2}+\gamma_{13}X_{1}+\delta_{2}$$ (12)

where $\alpha_{02}=\frac{\beta_{02}+\gamma_{12}\beta_{11}}{1-\gamma_{12}\gamma_{21}},\;\alpha_{CY2}=\frac{\beta_{CY2}+\gamma_{12}\beta_{CY1}}{1-\gamma_{12}\gamma_{21}},\;\alpha_{22}=\frac{\beta_{22}}{1-\gamma_{12}\gamma_{21}}$, and $\gamma_{13}=\frac{\gamma_{12}\beta_{11}}{1-\gamma_{12}\gamma_{21}}$. Formulas (3) and (12) can be written as

$$\left[\begin{array}{cc} 1 & -\gamma_{12}\\ 0 & 1 \end{array}\right]\left[\begin{array}{c} Y_{1}\\ Y_{2} \end{array}\right]=[\begin{array}{cccc} \beta_{01} & \beta_{CY1} & \beta_{11} & 0\\ \alpha_{02} & \alpha_{CY2} & \gamma_{13} & \alpha_{22} \end{array}]\left[\begin{array}{c} 1\\ C\\ X_{1}\\ X_{2} \end{array}\right]+[\begin{array}{c} \varepsilon_{1}\\ \delta_{2} \end{array}]$$

which can be represented as $\Gamma Y=BW+{\left[\begin{array}{cc} \varepsilon_{1} & E_{2} \end{array}\right]}^{T}$, where $Y={\left[Y_{1}\;Y_{2}\right]}^{T},\Gamma =[\begin{array}{cc} 1 & -\gamma_{21}\\ 0 & 1 \end{array}]$, $W={\left[\begin{array}{cccc} 1 & C & X_{1} & X_{2} \end{array}\right]}^{T}$, and $B=[\begin{array}{cccc} \beta_{01} & \beta_{CY1} & \beta_{11} & 0\\ \alpha_{02} & \alpha_{CY2} & \gamma_{13} & \alpha_{22} \end{array}].$ We assume $\left[\begin{array}{cc} \varepsilon_{1} & \delta_{2} \end{array}\right]$ follow multi-normal distribution. The maximum likelihood estimation of γ_12_ can be represented as

$${\left[\hat{\beta_{01}}\hat{\beta_{CY1}}\hat{\gamma_{21}}\right]}^{T}={\left(X^{T}(I_{n}-\hat{k}M_{w})X\right)}^{-1}X^{T}(I_{n}-\hat{k}M_{w})Y_{1}$$ (13)

where $X=[\begin{array}{cccc} 1 & C & X_{1} & Y_{2} \end{array}],M_{w}=I_{n}-W{\left(W^{T}W\right)}^{-1}W^{T},W=[\begin{array}{cccc} 1 & C & X_{1} & X_{2} \end{array}]$, and $\hat{k}$ is an eigenvalue of the matrix ${\left(Y^{T}M_{w}Y\right)}^{-1/2}Y^{T}M_{c}Y{\left(Y^{T}M_{w}Y\right)}^{-1/2}$, where $Y=[Y_{1}Y_{2}]$, $M_{C}=I_{n}-C^{*}{\left({C^{*}}^{T}C^{*}\right)}^{-1}{C^{*}}^{T}$, and $C^{*}=[\begin{array}{ccc} 1 & C & X_{1} \end{array}]$.

### Simulations

We assessed the robustness and accuracy of the proposed bidirectional methods using simulations. For each simulated dataset, we applied the traditional ratio and LIML methods and the proposed BiRatio and BiLIML methods. In each scenario, SNPs X_1_ and X_2_ were simulated with a minor allele frequency of 0.3, and the frequencies are assumed to be in Hardy-Weinberg proportions. The values of β_11_ and β_22_ were set to 1 or 2 to represent strong IVs and set to 0.02 or 0.05 to represent weak IVs. Also, in each scenario, the confounder C was generated from a normal distribution with mean 1 and unit variance. The regression coefficient of confounder C on Y_1_ and Y_2_, β_cy1_ and β_cy2_, respectively, were set to 0.3. The intercept values β_01_ and β_02_ were set to 1. The errors ε_1_ and ε_2_ were simulated from a standard normal distribution in each scenario. The datasets were generated with different numbers of strong IVs ranging from 1 to 20 and different numbers of weak IVs ranging from 1 to 100. For each scenario, we performed simulations with 1000 replicates.

*Simulation scenario 1—*the standard UMR model: We simulated data using the unidirectional model in Fig 1 with Formulas (1) and (4), in which the X_1_ is the IV for estimating the causal effect of Y_1_ on Y_2_ and the X_2_ is the IV for estimating the causal effect of Y_2_ on Y_1_. Because it is a unidirectional model, there is no causal effect of Y_2_ on Y_1_. The values of β_11_ and β_22_ were set to 1 when one strong IV was used; set to 2

when 20 strong IVs were used; set to 0.02 when 20 weak IVs were used; and set to 0.05 when 100 weak IVs were used. The purpose of this simulation was to confirm that the proposed BiRatio and BiLIML methods are appropriate for analyzing data even when the underlying model is unidirectional.

*Simulation scenario 2—*the BMR model: Outcomes Y_1_ and Y_2_ were generated using Formulas (5) and (6) in the BMR model. The values of β_11_ and β_22_ were set to 1 when one strong IV was used; and set to 2 when 5, 10, or 20 strong IVs were used. The purpose of this simulation was to evaluate the accuracy of proposed methods when simulated data have a bidirectional causal relationship, and instrumental variables are strong.

*Simulation scenario 3—*the BMR model: The outcomes Y_1_ and Y_2_ were generated using Formulas (5) and (6) in the BMR model. The values of β_11_ and β_22_ were set to 0.02 when 1, 5, 10, or 20 weak IVs were used; and set to 0.05 when 100 weak IVs were used. The purpose of this simulation was to evaluate the accuracy of proposed bidirectional methods when simulated data have a bidirectional causal relationship and instrumental variables are weak.

*Measures of Performance*: We evaluated the bias in estimation for a range of positive and negative values of γ_12_ from -1.9 to 1.9 for simulation scenario 1 and a range of positive and negative values of γ_12_ and γ_21_ from -1.9 to 1.9 for simulation scenarios 2 and 3. The mean value of F-statistics of regressing each X_1_ on Y_1_ over 1000 replicates was used to assess the strength of X_1_ as an IV for γ_12_ estimation. Similarly, the mean value of F-statistics of regressing each X_2_ on Y_2_ over 1000 replicates was used to assess the strength of X_2_ as an IV for γ_21_ estimation. The proposed methods’ performances were evaluated using the following metrics: 1) We determined the median value of estimated γ_12_ and γ_21_ from 1000 replicates. 2) We calculated the median absolute bias (MAB) $=Median(|\hat{\gamma_{12}}-\gamma_{12}|)\;and\;Median(|\hat{\gamma_{21}}-\gamma_{21}|)$: In each replicate, we calculated the absolute value of the difference between estimated γ_12_ or γ_21_ and their corresponding true value as absolute bias. The MAB is the median value of the 1000 absolute biases from 1000 replicates. 3) We calculated the relative median absolute bias (RMAB) $=\frac{MAB_{\gamma_{12}}}{\left|\gamma_{12}\right|}\;and\;\frac{MAB_{\gamma_{21}}}{\left|\gamma_{21}\right|}$. When the simulated data are from the UMR model, the true γ_21_ is 0, and the RMAB of estimated γ_21_ is not defined. We used the R programming language for all simulations and analyses. The mr_ivw function from the R package MendelianRandomization, version 0.6.0, with default settings [40] was used for Ratio and BiRatio methods. The LIML function from the R package ivmodel, version 1.81, with default settings [41] was used for both LIML and BiLIML methods. We have created a BiMR statistical package in R, which contains the simulation code and functions to estimate the bidirectional causal effects. The package can be installed from github: https://github.com/JinhaoZou/BiMR.

## Results

### Simulations

*Simulation scenario 1*: In this scenario, 1000 replicates of the data from 1000 individuals were simulated using the UMR model (Fig 1) with γ_12_ values from -1.9 to 1.9. The causal effects were estimated using the four methods (ratio, BiRatio, LIML, and BiLIML). In Table 1, we present the results in four sections using different numbers and strengths of IVs: 1 strong IV, 20 strong IVs, 20 weak IVs, and 100 weak IVs. For each section, the first column represents the true simulated values of γ_12_, the second column reports the F-statistics quantifying the strengths of IVs, and the subsequent columns represent the measures of performance (median, MAB, and RMAB) for the four methods. When strong IVs were used, all four methods provided accurate estimations. For example, when 1 or 20 strong IVs were used, the estimated MAB for the four methods ranged from 0.00 to 0.04, and the estimated RMAB for the four methods ranged from 0% to 2%. The LIML and BiLIML methods provided more accurate estimations than the ratio and BiRatio methods when weak IVs were used. For example, when 100 weak IVs were used, the estimated MAB and RMAB for the ratio and BiRatio methods ranged from 0.02 to 0.21 and 3% to 7%, respectively, while the estimated MAB and RMAB for LIML and BiLIML methods ranged from 0.02 to 0.03, and 1% and 3%, respectively.

**Table 1:** Simulation scenario 1: The unidirectional Mendelian randomization model is used. Parameter estimates are based on 1000 replicates.

	F-stat	Ratio			BiRatio			LIML			BiLML		
		Median	MAB	RMAB	Median	MAB	RMAB	Median	MAB	RMAB	Median	MAB	RMAB
**Strong IV (1)†**													
γ_12_ = −1.9 γ_21_ = 0	6231.40	-1.90	0.02	1%	-1.90	0.01	1%	-1.90	0.02	1%	-1.90	0.01	1%
	262.56	0.00	0.02	-	0.00	0.01	-	0.00	0.02	-	0.00	0.01	-
γ_12_ = −0.9 γ_21_ = 0	6238.96	-0.90	0.02	2%	-0.9	0.01	1%	-0.90	0.02	2%	-0.90	0.01	1%
	1174.58	0.00	0.02	-	0.00	0.01	-	0.00	0.02	-	0.00	0.01	-
γ_12_ = 0.9 γ_21_ = 0	6226.88	0.90	0.02	2%	0.90	0.01	1%	0.90	0.02	2%	0.90	0.01	1%
	741.36	0.00	0.02	-	0.00	0.01	-	0.00	0.02	-	0.00	0.01	-
γ_12_ = 1.9 γ_21_ =0	6224.88	1.90	0.02	1%	1.90	0.01	1%	1.90	0.02	1%	1.90	0.01	1%
	206.55	0.00	0.02	-	0.00	0.01	-	0.00	0.02	-	0.00	0.01	-
**Strong IVs (20)†**													
γ_12_ = −1.9 γ_21_ = 0	53.35	-1.90	0.02	1%	-1.90	0.00	0%	-1.90	0.02	1%	-1.90	0.00	0%
	11.80	-0.03	0.04	-	0.00	0.00	-	0.00	0.02	-	0.00	0.00	-
γ_12_ = −0.9 γ_21_ = 0	53.78	-0.90	0.02	2%	-0.90	0.00	0%	-0.90	0.02	2%	-0.90	0.00	0%
	29.74	-0.02	0.02	-	0.00	0.00	-	0.00	0.02	-	0.00	0.00	-
γ_12_ = 0.9 γ_21_ = 0	53.56	0.90	0.02	2%	0.90	0.00	0%	0.90	0.02	2%	0.90	0.00	0%
	28.84	0.02	0.02	-	0.00	0.00	-	0.00	0.02	-	0.00	0.00	-
γ_12_ = 1.9 γ_21_ = 0	53.99	1.90	0.02	1%	1.90	0.00	0%	1.90	0.02	1%	1.90	0.00	0%
	12.18	0.03	0.03	-	0.00	0.00	-	0.00	0.02	-	0.00	0.00	-
**Weak IVs (20)†**													
γ_12_ = −1.9 γ_21_ = 0	3.28	-1.67	0.23	12%	-1.66	0.24	13%	-1.91	0.10	5%	-1.91	0.10	5%
	2.54	-0.28	0.28	-	-0.27	0.27	-	0.00	0.10	-	0.01	0.10	-
γ_12_ = −0.9 γ_21_ = 0	3.58	-0.65	0.25	28%	-0.65	0.25	28%	-0.88	0.10	11%	-0.89	0.10	11%
	7.98	-0.02	0.09	-	-0.01	0.09	-	0.01	0.10	-	0.01	0.10	-
γ_12_ = 0.9 γ_21_ = 0	3.12	1.14	0.24	27%	1.15	0.25	28%	0.90	0.10	11%	0.91	0.10	11%
	1.68	0.30	0.30	-	0.30	0.30	-	-0.01	0.11	-	-0.01	0.11	-
γ_12_ = 1.9 γ_21_ = 0	3.50	2.14	0.24	13%	2.15	0.25	13%	1.90	0.10	5%	1.91	0.10	5%
	1.38	0.27	0.27	-	0.26	0.26	-	0.01	0.09	-	0.01	0.09	-
**Weak IVs (100)†**													
γ_12_ = −1.9 γ_21_ = 0	7.23	-1.84	0.06	3%	-1.84	0.06	3%	-1.90	0.03	2%	-1.90	0.02	1%
	2.77	-0.19	0.19	-	-0.08	0.08	-	0.00	0.03	-	0.00	0.02	-
γ_12_ = −0.9 γ_21_ = 0	7.15	-0.84	0.06	7%	-0.84	0.06	7%	-0.90	0.03	3%	-0.90	0.02	2%
	5.86	-0.09	0.09	-	0.00	0.02	-	0.00	0.03	-	0.00	0.02	-
γ_12_ = 0.9 γ_21_ = 0	6.88	0.96	0.06	7%	0.96	0.06	7%	0.90	0.03	3%	0.90	0.02	2%
	3.65	0.17	0.17	-	0.11	0.11	-	0.00	0.03	-	0.00	0.02	-
γ_12_ = 1.9 γ_21_ = 0	7.18	1.96	0.06	3%	1.96	0.06	3%	1.90	0.03	2%	1.90	0.02	1%
	2.07	0.21	0.21	-	0.13	0.13	-	0.00	0.03	-	0.00	0.02	-

BiRatio = bidirectional ratio method; BiLIML = limited information maximum likelihood method; IVs = instrumental variables; LIML = limited information maximum likelihood method. Median is the median value of estimated causal effect among 1000 replicates. MAB is the median of absolute bias of each estimation among 1000 replicates. RMAB is the relative median of absolute bias of each estimation among 1000 replicates.

†shows the number of instrumental variables used for generating dataset.

**Fig 3:**
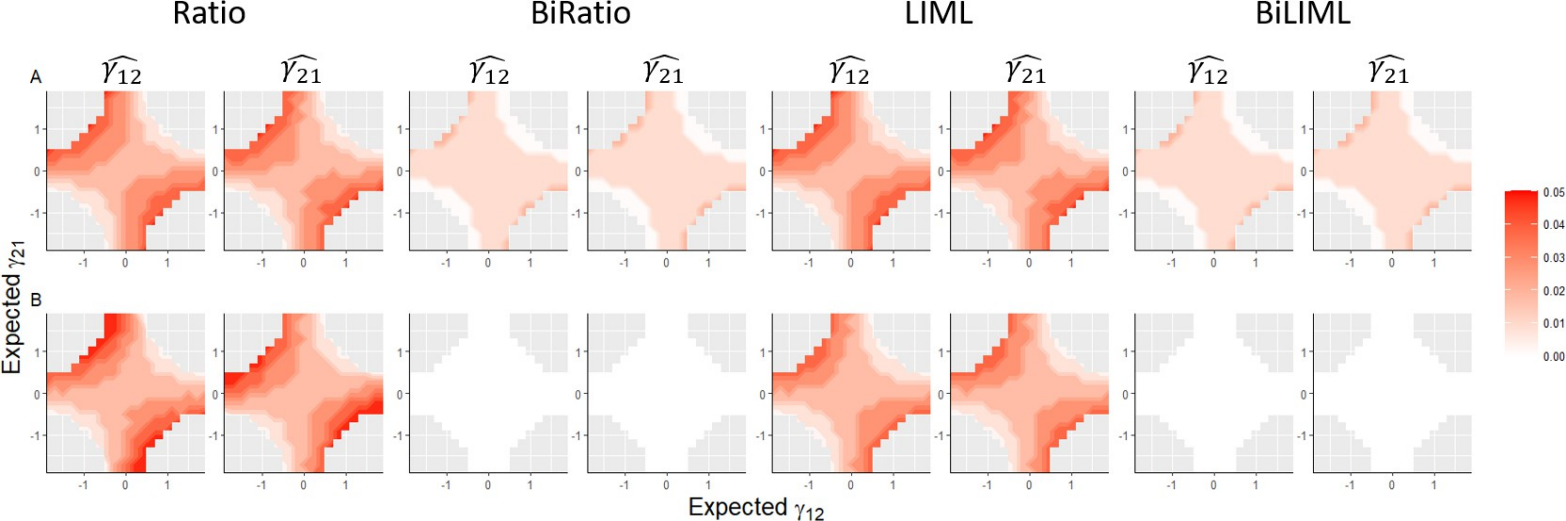
Median of absolute bias (MAB) of bidirectional causal effect estimations for simulation scenario 2: Simulation using the bidirectional Mendelian randomization model and strong instrumental variables (IVs). Parameter estimations are based on 1000 simulation replicates. A: MAB of estimations using one strong IV. B: MAB of estimations using 20 strong IVs. The color bar shows the range of MAB. BiRatio = bidirectional ratio method; BiLIML = limited information maximum likelihood method; LIML = limited information maximum likelihood method.

*Simulation scenario 2*: For this scenario, 1000 replicates of the data from 1000 individuals were simulated using the BMR model (Fig 2) with strong IVs ranging from 1 to 20. The expected values of γ_12_ and γ_21_ values were set ranging from -1.9 to 1.9. The causal effects were estimated using the four methods (ratio, BiRatio, LIML, and BiLIML). In Table 2 and Fig 3, the results are presented in four sections using different numbers of strong IVs: 1, 5, 10, and 20. Similar to Table 1, for each section, the first column represents the true simulated values of γ_12_ and γ_12_, the second column reports the F-statistics quantifying the strengths of IVs, and the subsequent columns represent the measures of performance (median, MAB, and RMAB) for the four methods. When strong IV was used, and the true γ_12_ and γ_21_ had opposite directions, the BiRatio and BiLIML methods provided more accurate estimations than the ratio and LIML methods (Table 2 and Fig 3). For example, when γ_12_ = -1.9 and γ_21_ = 0.5, the estimated RMAB for γ_12_ and γ_21_ estimation using the ratio and LIML methods were 3% and 8%, respectively, while the estimated RMAB for γ_12_ and γ_21_ estimation using the BiRatio and BiLIML methods were 1% and 4%, respectively. When multiple strong IVs were used, and the true γ_12_ and γ_21_ values had opposite signs, the BiRatio and BiLIML methods provided more accurate estimations compared to the naïve application of the standard ratio and LIML methods (Fig 3). For example, when 20 strong IVs were used, the estimated MAB and RMAB for the BiRatio and BiLIML methods were 0 and 0%, respectively, while the MAB and RMAB for the standard ratio and LIML ranged from 0.04 to 0.07 and 2% to 14%, respectively (Table 2).

**Table 2:** Simulation scenario 2 with strong instrumental variables (IVs): The bidirectional Mendelian randomization model is used. Parameter estimates are based on 1000 replicates.

	F-stat	Ratio			BiRatio			LIML			BiLIML		
		Median	MAB	RMAB	Median	MAB	RMAB	Median	MAB	RMAB	Median	MAB	RMAB
**Strong IV (1)†**													
*γ*_12_ = −1.9 *γ*_21_ = −0.5	3179.44	-1.90	0.00	0%	-1.90	0.00	0%	-1.90	0.00	0%	-1.90	0.00	0%
	262.58	-0.50	0.00	0%	-0.50	0.00	0%	-0.50	0.00	0%	-0.50	0.00	0%
*γ*_12_ = −1.9 *γ*_21_ = 0.5	1699.74	-1.90	0.05	3%	-1.90	0.02	1%	-1.90	0.05	3%	-1.90	0.02	1%
	262.47	0.50	0.04	8%	0.50	0.02	4%	0.50	0.04	8%	0.50	0.02	4%
*γ*_12_ = −0.9 *γ*_21_ = 0.9	744.14	-0.90	0.04	4%	-0.90	0.02	2%	-0.90	0.04	4%	-0.90	0.02	2%
	1169.82	0.90	0.04	4%	0.90	0.01	1%	0.90	0.04	4%	0.90	0.01	1%
*γ*_12_ = 0.9 *γ*_21_ = −0.9	1168.08	0.90	0.04	4%	0.90	0.02	2%	0.90	0.04	4%	0.90	0.02	2%
	746.06	-0.90	0.04	4%	-0.90	0.02	2%	-0.90	0.04	4%	-0.90	0.02	2%
**Strong IVs (5)†**													
*γ*_12_ = −1.9 *γ*_21_ = −0.5	191.45	-1.90	0.00	0%	-1.90	0.00	0%	-1.90	0.00	0%	-1.90	0.00	0%
	46.25	-0.50	0.00	0%	-0.50	0.00	0%	-0.50	0.00	0%	-0.50	0.00	0%
*γ*_12_ = −1.9 *γ*_21_ = 0.5	190.21	-1.89	0.04	2%	-1.90	0.00	0%	-1.90	0.04	2%	-1.90	0.00	0%
	46.08	0.49	0.04	8%	0.50	0.00	0%	0.50	0.04	8%	0.50	0.00	0%
*γ*_12_ = −0.9 *γ*_21_ = 0.9	123.57	-0.89	0.04	4%	-0.90	0.00	0%	-0.90	0.04	4%	-0.90	0.00	0%
	126.36	0.90	0.04	4%	0.90	0.00	0%	0.90	0.04	4%	0.90	0.00	0%
*γ*_12_ = 0.9 *γ*_21_ = −0.9	126.48	0.90	0.04	4%	0.90	0.00	0%	0.90	0.04	4%	0.90	0.00	0%
	123.19	-0.89	0.04	4%	-0.90	0.00	0%	-0.90	0.04	4%	-0.90	0.00	0%
**Strong IVs (10)†**													
*γ*_12_ = −1.9 *γ*_21_ = −0.5	87.98	-1.90	0.00	0%	-1.90	0.00	0%	-1.90	0.00	0%	-1.90	0.00	0%
	22.85	-0.50	0.00	0%	-0.50	0.00	0%	-0.50	0.00	0%	-0.50	0.00	0%
*γ*_12_ = −1.9 *γ*_21_ = 0.5	87.86	-1.89	0.04	2%	-1.90	0.00	0%	-1.90	0.04	2%	-1.90	0.00	0%
	23.32	0.47	0.05	10%	0.50	0.00	0%	0.50	0.04	8%	0.50	0.00	0%
*γ*_12_ = −0.9 *γ*_21_ = 0.9	59.24	-0.89	0.04	4%	-0.90	0.00	0%	-0.90	0.04	4%	-0.90	0.00	0%
	59.53	0.89	0.04	4%	0.90	0.00	0%	0.90	0.04	4%	0.90	0.00	0%
*γ*_12_ = 0.9 *γ*_21_ = −0.9	59.18	0.89	0.04	4%	0.90	0.00	0%	0.90	0.04	4%	0.90	0.00	0%
	59.16	-0.88	0.04	4%	-0.90	0.00	0%	-0.90	0.04	4%	-0.90	0.00	0%
**Strong IVs (20)†**													
*γ*_12_ = −1.9 *γ*_21_ = −0.5	42.33	-1.90	0.00	0%	-1.90	0.00	0%	-1.90	0.00	0%	-1.90	0.00	0%
	12.18	-0.50	0.00	0%	-0.50	0.00	0%	-0.50	0.00	0%	-0.50	0.00	0%
*γ*_12_ = −1.9 *γ*_21_ = 0.5	42.54	-1.88	0.04	2%	-1.90	0.00	0%	-1.90	0.04	2%	-1.90	0.00	0%
	12.18	0.43	0.07	14%	0.50	0.00	0%	0.50	0.04	8%	0.50	0.00	0%
*γ*_12_ = −0.9 *γ*_21_ = 0.9	29.05	-0.87	0.05	6%	-0.90	0.00	0%	-0.90	0.04	4%	-0.90	0.00	0%
	29.12	0.87	0.04	4%	0.90	0.00	0%	0.90	0.04	4%	0.90	0.00	0%
*γ*_12_ = 0.9	29.46	0.87	0.04	4%	0.90	0.00	0%	0.90	0.04	4%	0.90	0.00	0%
*γ*_21_ = −0.9	29.35	-0.86	0.05	6%	-0.90	0.00	0%	-0.90	0.04	4%	-0.90	0.00	0%

BiRatio = bidirectional ratio method; BiLIML = limited information maximum likelihood method; IVs = instrumental variables; LIML = limited information maximum likelihood method. Median is the median value of estimated causal effect among 1000 replicates. MAB is the median of absolute bias of each estimation among 1000 replicates. RMAB is the relative median of absolute bias of each estimation among 1000 replicates.

†shows the number of instrumental variables used for generating dataset.

**Fig 4:**
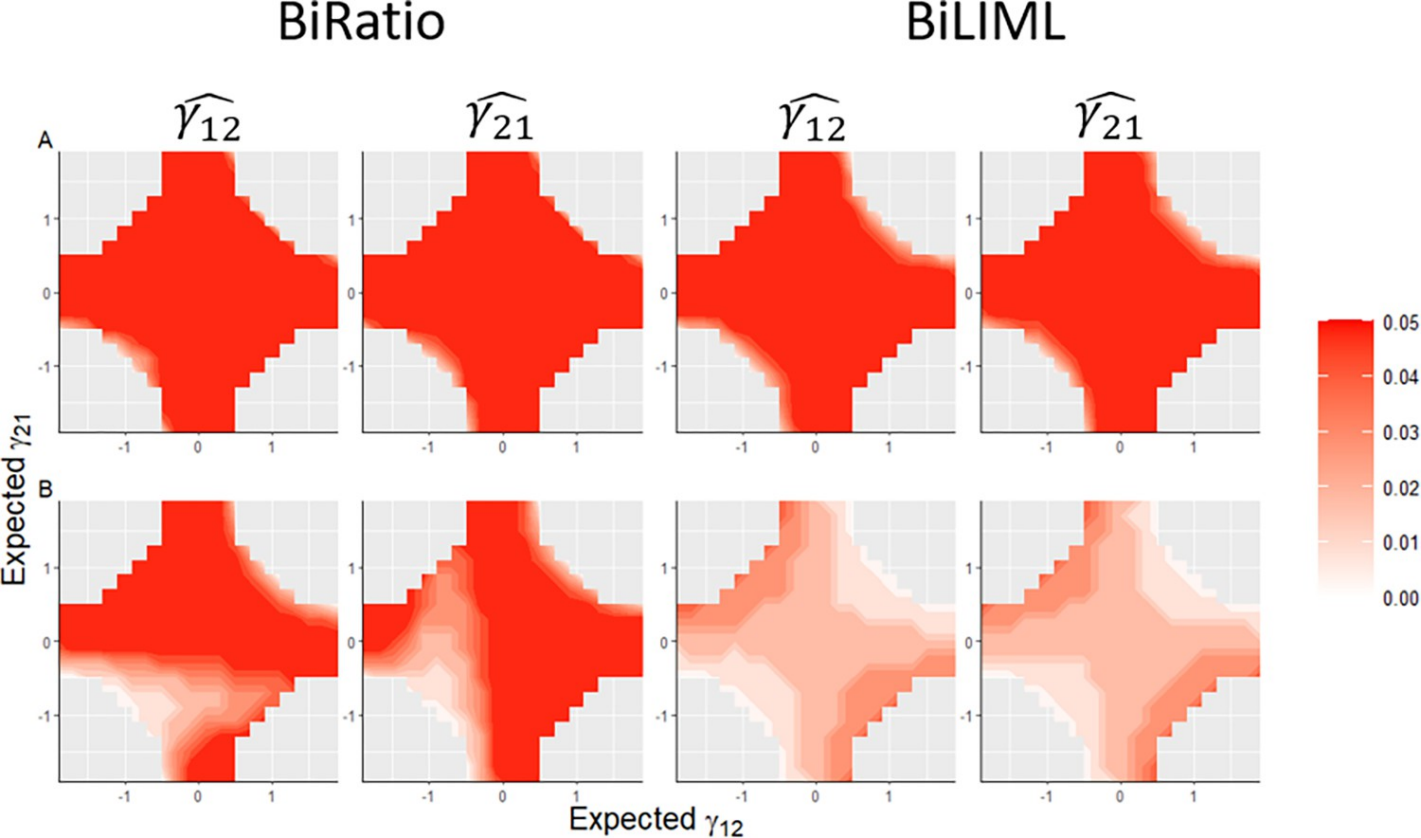
Median of absolute bias (MAB) of bidirectional causal effect estimations for simulation scenario 3: Simulation using the bidirectional Mendelian randomization model and weak instrumental variables (IVs). Parameter estimations are based on 1000 simulation replicates. A: MAB of estimations using 20 weak IVs. B: MAB of estimations using 100 weak IVs. The color bar for each figure shows the range of the MAB. BiRatio = bidirectional ratio method; BiLIML = limited information maximum likelihood method.

*Simulation scenario 3*: Because the BiRatio and BiLML methods performed identically when strong IVs were used, to evaluate the performance of these two methods when only weak IVs are available, we simulated 1000 replicates of the data with 1000 individuals using the BMR model with weak IVs ranging from 1 to 100 (Table 3 and Fig 4). The expected value of γ_12_ and γ_21_ values were set, ranging from -1.9 to 1.9. In Table 3, we present the results in five sections using the different numbers of weak IVs: 1, 5, 10, 20, and 100. When multiple weak IVs were used, BiLIML provided more accurate estimations than the BiRatio method (Table 3 and Fig 4). For example, when 100 weak IVs were used and the directions of γ_12_ and γ_21_ are opposite, the estimated MAB for the BiRatio method ranged from 0.03 to 0.20, while the estimated MAB for the BiLIML method ranged from 0.03 to 0.04. Also, the estimated RMAB for the BiRatio method ranged from 3% to 30%, while the estimated RMAB for the BiLIML method ranged from 3% to 8% (Table 3).

**Table 3:** Simulation scenario 3 with weak instrumental variables: The simulation model is the bidirectional Mendelian randomization model. Parameter estimates are based on 1000 replicates.

	F-stat	BiRatio			BiLIML		
		Median	MAB	RMAB	Median	MAB	RMAB
**Weak IV (1)†**							
*γ*_12_ = −1.9 *γ*_21_ = −0.5	7.10	-1.90	0.02	1%	-1.90	0.02	1%
	2.85	-0.51	0.02	4%	-0.51	0.02	4%
*γ*_12_ = −1.9 *γ*_21_ = 0.5	2.19	-1.59	0.71	37%	-1.59	0.71	37%
	2.70	0.24	0.72	144%	0.24	0.72	144%
*γ*_12_ = −0.9 *γ*_21_ = 0.9	1.71	-0.54	0.56	62%	-0.54	0.56	62%
	9.98	0.89	0.72	80%	0.89	0.72	80%
*γ*_12_ = 0.9 *γ*_21_ = −0.9	10.12	0.91	0.76	84%	0.91	0.76	84%
	1.70	-0.53	0.58	64%	-0.53	0.58	64%
**Weak IVs (5)†**							
*γ*_12_ = −1.9 *γ*_21_ = −0.5	7.08	-1.89	0.01	1%	-1.90	0.01	1%
	2.68	-0.51	0.01	2%	-0.50	0.01	2%
*γ*_12_ = −1.9 *γ*_21_ = 0.5	2.26	-1.35	0.55	29%	-1.91	0.36	19%
	2.69	0.01	0.51	102%	0.47	0.38	76%
*γ*_12_ = −0.9 *γ*_21_ = 0.9	1.71	-0.39	0.51	57%	-0.85	0.34	38%
	8.97	0.88	0.33	37%	0.89	0.36	40%
*γ*_12_ = 0.9 *γ*_21_ = −0.9	9.44	0.89	0.33	37%	0.93	0.36	40%
	1.79	-0.38	0.52	58%	-0.90	0.35	39%
**Weak IVs (10)†**							
*γ*_12_ = −1.9 *γ*_21_ = −0.5	6.70	-1.89	0.01	1%	-1.90	0.01	1%
	2.71	-0.51	0.01	2%	-0.50	0.01	2%
*γ*_12_ = −1.9 *γ*_21_ = 0.5	2.15	-1.33	0.57	30%	-1.89	0.28	15%
	2.58	-0.01	0.51	102%	0.53	0.29	58%
*γ*_12_ = −0.9 *γ*_21_ = 0.9	1.79	-0.36	0.54	60%	-0.90	0.25	28%
	8.97	0.87	0.23	26%	0.91	0.25	28%
*γ*_12_ = 0.9 *γ*_21_ = −0.9	8.78	0.86	0.24	27%	0.89	0.25	28%
	1.76	-0.37	0.53	59%	-0.88	0.24	27%
**Weak IVs (20)†**							
*γ*_12_ = −1.9 *γ*_21_ = −0.5	6.47	-1.89	0.01	1%	-1.90	0.00	0%
	2.63	-0.51	0.01	2%	-0.50	0.01	2%
*γ*_12_ = −1.9 *γ*_21_ = 0.5	2.22	-1.32	0.58	31%	-1.89	0.20	11%
	2.53	-0.04	0.54	108%	0.50	0.20	40%
*γ*_12_ = −0.9 *γ*_21_ = 0.9	1.68	-0.35	0.55	61%	-0.91	0.18	20%
	8.06	0.86	0.17	19%	0.89	0.19	21%
*γ*_12_ = 0.9 *γ*_21_ = −0.9	7.92	0.88	0.16	18%	0.91	0.18	20%
	1.58	-0.36	0.54	60%	-0.92	0.19	21%
**Week IVs (100)†**							
*γ*_12_ = −1.9 *γ*_21_ = −0.5	7.60	-1.90	0.00	0%	-1.90	0.00	0%
	2.67	-0.50	0.00	0%	-0.50	0.00	0%
*γ*_12_ = −1.9 *γ*_21_ = 0.5	4.99	-1.72	0.18	9%	-1.90	0.04	2%
	2.75	0.35	0.15	30%	0.50	0.04	8%
*γ*_12_ = −0.9 *γ*_21_ = 0.9	3.39	-0.70	0.20	22%	-0.90	0.03	3%
	6.31	0.90	0.03	3%	0.90	0.03	3%
*γ*_12_ = 0.9 *γ*_21_ = −0.9	5.97	0.89	0.04	4%	0.90	0.03	3%
	3.51	-0.70	0.20	22%	-0.90	0.03	3%

BiRatio = bidirectional ratio method; BiLIML = limited information maximum likelihood method; IVs = instrumental variables. Median is the median value of estimated causal effect among 1000 replicates. MAB is the median of absolute bias of each estimation among 1000 replicates. RMAB is the relative median of absolute bias of each estimation among 1000 replicates.

†shows the number of instrumental variables used for generating dataset.

### Bidirectional causal relationship between BMI and FG: MESA cohort

We applied the four methods (ratio, BiRatio, LIML, BiLIML) to investigate a possible bidirectional causal relationship between BMI and FG using the data from the MESA cohort, which contains 47871 SNPs and 5764 individuals. We excluded 13 individuals due to missing data for BMI and FG. We also excluded 300 outlier individuals whose BMI was greater than 45 and whose FG was greater than 160. To reduce the confounding effects of race/ethnicity on SNPs, exposures, and outcomes, we separated the 5451 individuals into four racial/ethnic groups: group 1 with 2235 White/Caucasian individuals, group 2 with 669 Chinese American individuals, group 3 with 1358 African American individuals, and group 4 with 1189 Hispanic individuals. We excluded 40, 41, 46, and 11 individuals from these four groups, respectively, due to the close family relationships between individuals (kinship coefficients > 0.1) [42]. Also, SNPs with minor allele frequency less than 0.05, located within non-autosomes and having linkage disequilibrium (LD) over 0.1, were removed from each group separately. Thus, the study samples included 2195 individuals with 31039 SNPs, 628 individuals with 28214 SNPs, 1312 individuals with 36931 SNPs, and 1081 individuals with 32729 SNPs for groups 1, 2, 3, and 4, respectively.

In each group, we performed genetic association to investigate relationships between BMI and FG, adjusting for sex, age, and the first 10 principal components (PCs). The top 20 SNPs associated only with BMI and not directly associated with FG given BMI, sex, age, and first 10 PC were selected as IVs for BMI. Similarly, the top 20 SNPs associated only with FG and not directly associated with BMI were selected as IVs for FG. Analyses of the data using the BiLIML method showed bidirectional causal relationships between BMI and FG in all four racial/ethnic groups (Table 4). For example, in group 1, the causal effect estimated by BiLIML of BMI on FG was 0.7003 (95%CI: 0.3517–1.0489; *p* = 8.43×10^−5^), which means that a BMI increase of 1 kg/m^2^ can result in an FG increase of 0.7003 mg/dL. In the same group, the causal effect estimated by BiLIML of FG on BMI was 0.1041 (95% CI: 0.0441–0.1640; *p* = 6.79×10^−4^), which indicates that an FG increase of 1 mg/dL can result in a BMI increase of 0.1041 kg/m^2^.

**Table 4:** The bidirectional causal effects estimation between body mass index and fasting glucose.

		Causal effect of BMI on FG				Causal effect of FG on BMI			
Race	Method	Variance of BMI explained	F-statistic	Estimation (95% CI)	P-value	Variance of FG explained	F-statistic	Estimation (95% CI)	P-value
**White/Caucasian (2195 individuals)**	**Ratio**	10.40%	13.30	0.7327 (0.3419–1.1236)	2.38*10^−4^	6.29%	14.00	0.1019 (0.0210–0.1827)	1.35*10^−2^
	**BiRatio**	10.40%	13.30	0.6982 (0.3141–1.0822)	3.66*10^−4^	6.29%	14.00	0.1036 (0.0261–0.1810)	8.77*10^−3^
	**LIML**	10.40%	13.30	0.7385 (0.3842–1.0927)	4.51*10^−5^	6.29%	14.00	0.1159 (0.0538–0.1780)	2.60*10^−4^
	**BiLIML**	10.40%	13.30	0.7003 (0.3517–1.0489)	8.43*10^−5^	6.29%	14.00	0.1041 (0.0441–0.1640)	6.79*10^−4^
**Chinese American (628 individuals)**	**Ratio**	23.55%	12.48	0.8004 (-0.1558–1.7565)	1.01*10^−1^	24.94%	12.29	0.0669 (0.0188–0.1150)	6.45*10^−3^
	**BiRatio**	23.55%	12.48	1.0045 (0.0522–1.9567)	3.87*10^−2^	24.94%	12.29	0.0742 (0.0263–0.1222)	2.41*10^−3^
	**LIML**	23.55%	12.48	0.9257 (0.1456–1.7058)	2.01*10^−2^	24.94%	12.29	0.0675 (0.0301–0.1049)	4.24*10^−4^
	**BiLIML**	23.55%	12.48	0.9344 (0.2016–1.6671)	1.26*10^−2^	24.94%	12.29	0.0746 (0.0384–0.1109)	6.08*10^−6^
**Black/African American** **(1312 individuals)**	**Ratio**	15.00%	13.38	0.6913 (0.1680–1.2146)	9.62*10^−3^	15.50%	13.45	0.0394 (-0.0196–0.0984)	1.90*10^−1^
	**BiRatio**	15.00%	13.38	0.8823 (0.3648–1.3999)	8.34*10^−4^	15.50%	13.45	0.0482 (-0.0077–0.1041)	9.09*10^−2^
	**LIML**	15.00%	13.38	0.8519 (0.3991–1.3046)	2.33*10^−4^	15.50%	13.45	0.0374 (-0.0136–0.0883)	1.50*10^−1^
	**BiLIML**	15.00%	13.38	1.0063 (0.5451–1.4675)	2.03*10^−5^	15.50%	13.45	0.0494 (0.0015–0.0973)	4.32*10^−2^
**Hispanic** **(1081 individuals)**	**Ratio**	17.24%	14.97	0.7796 (0.0321–1.5271)	4.09*10^−2^	12.21%	13.21	0.0992 (0.0386–0.1599)	1.33*10^−3^
	**BiRatio**	17.24%	14.97	0.5772 (-0.1413–1.2956)	1.15*10^−1^	12.21%	13.21	0.0605 (0.0001–0.1209)	4.98*10^−2^
	**LIML**	17.24%	14.97	0.8433 (0.3110–1.3756)	1.93*10^−3^	12.21%	13.21	0.0966 (0.0454–0.1479)	2.28*10^−4^
	**BiLIML**	17.24%	14.97	0.7094 (0.2014–1.2174)	6.24*10^−3^	12.21%	13.21	0.0730 (0.0226–0.1233)	4.56*10^−3^

BiRatio = bidirectional ratio method; BiLIML = limited information maximum likelihood method; BMI = body mass index; FG = fasting glucose; LIML = limited information maximum likelihood method.

## Discussion

The Mendelian randomization model is widely used to estimate the causal effects of exposures on outcomes in observational studies. In general, the MR methods are used for the estimation of unidirectional causal effects; however, many phenotypes may have bidirectional causal relationships. Typically, bidirectional causal effects have been estimated using two unidirectional MR models, one for each causal direction [32,43,44]. However, such an approach ignores the bidirectional feedback loop between two phenotypes, leading to biased effect estimation. Therefore, in this manuscript, we proposed two novel approaches to estimate bidirectional causal effects using MR: BiRatio and BiLIML, extended versions of the standard ratio and LIML methods, respectively. We compared the performance of the two proposed methods with the naive application of UMR methods through extensive simulations involving varying numbers of strong and weak IVs. We used three measures to evaluate the accuracy of the proposed methods: median, MAB, and RMAB. Our simulation results showed that both the proposed BiRatio and BiLIML methods provided accurate estimations of causal effects even when the true causal relationship was unidirectional. Importantly, when the true causal relationship was bidirectional and strong IVs were used, both the proposed methods provided accurate causal effect estimates compared to the naïve application of ratio and LIML methods. The poor performance of the naïve application of ratio and LIML methods was more pronounced when the true bidirectional causal effects were in the opposite direction (opposite signs). Furthermore, when weak IVs were used, the BiLIML method performed better than the BiRatio method. Therefore, we recommend using the BiLIML method as the primary method for bidirectional causal effect estimation.

Because of the interdependence of obesity and diabetes, we hypothesized that there is a bidirectional relationship between obesity and diabetes. We applied the proposed methods to investigate the potential bidirectional relationship using the data from the Multi-Ethnic Study of Atherosclerosis cohort. We used body mass index (BMI) and fasting glucose (FG) as measures of obesity and type 2 diabetes, respectively. Because of the underlying biological differences among White/Caucasian, Chinese Americans, African Americans, and Hispanics, we performed separate analyses to investigate the causal relationships in these racial/ethnic subpopulations. The BiLIML method identified novel statistically significant bidirectional causal effects between BMI and FG in all the subpopulations. However, further studies are needed to understand the biological and functional mechanisms underlying the identified bidirectional relationship.

Our proposed BiRatio and BiLIML methods require similar assumptions and are subject to similar limitations as the standard MR-based ratio and LIML methods for valid causal inference. Besides the weak instrumental bias, the issue of selecting valid IVs is a common concern for MR-based methods because of the possible horizontal pleiotropy of IVs, underlying population stratification, and the winner’s curse. In our simulations, we generated the SNPs independently and simulated the data following the three assumptions of MR. However, selected SNPs as IVs may have horizontal pleiotropy in MR studies, where the IVs have an association with multiple traits independent of exposure [10]. Such associations might lead to a violation of the MR assumptions due to the existence of causal effects from selected SNPs on confounders or outcomes independent of exposure. Also, when the IVs are selected based on associations derived from a heterogeneous population, the instrumental variable SNPs may be erroneously selected due to underlying population stratification instead of their true association with the exposure. In addition, when the same sample is used for identifying IVs and using them for MR studies, the estimated SNP-exposure association may be biased upwards, an effect known as the “winner’s curse” in the literature [10,17,45]. It is suggested that selecting genetic variants as IVs based on their biological functions can reduce bias due to population stratification and the winner’s curse [10,19]. Most importantly, for MR-based methods, sensitivity analyses are essential [19].

Furthermore, our proposed methods are valid for continuous outcomes and one-sample datasets. Further research in bidirectional causal estimation using binary outcomes, summary statistics, and two-sample datasets is needed. Also, our model was designed to provide causal inference using cross-sectional data. Therefore, further extensions when the exposure or outcome variables are time-varying are of importance for future research.

In summary, we proposed two methods for bidirectional causal effect estimation that were shown to be accurate when the underlying model is unidirectional or bidirectional. Furthermore, applying the proposed methods to the MESA data provided preliminary evidence for the bidirectional causal effects between BMI and FG.

## Supporting information

S1 Fig Bidirectional Mendelian randomization model with transitional steps in the feedback loop. X_1_ denotes the instrumental variables (IVs) for Y_1_ and X_2_ denotes the IVs for Y_2_. The ε_1.1_, ε_1.2_, …, ε_2.1_, ε_2.2_,… are errors due to unobserved confounders at each transitional step. Of note, these transitional steps are not observed and only Y_1_ and Y_2_ are measured. The δ_1_ and δ_2_ are errors associated with Y_1_ and Y_2_, respectively, which includes both errors due to unobserved confounders as well as measurement errors.
(TIF)

S1 Appendix Derivation for bidirectional Mendelian randomization model. (PDF)
