# Supplementary figures and images for: Approaches to estimate bidirectional causal effects using Mendelian randomization with application to body mass index and fasting glucose
Source: PLoS One. 2024 Mar 8;19(3):e0293510. doi: 10.1371/journal.pone.0293510 (PMC10923437; doi:10.1371/journal.pone.0293510)

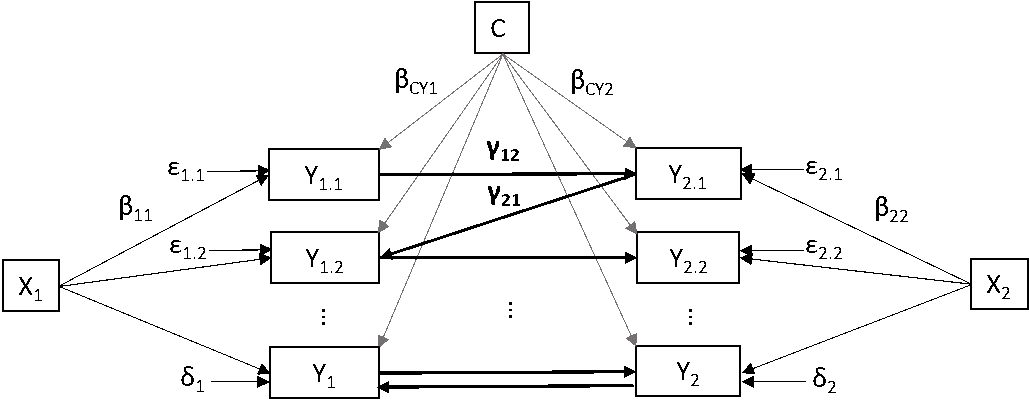

Supplement: S1 Fig — X1 denotes the instrumental variables (IVs) for Y1 and X2 denotes the IVs for Y2. The ε1.1, ε1.2, …, ε2.1, ε2.2,… are errors due to unobserved confounders at each transitional step. Of note, these transitional steps are not observed and only Y1 and Y2 are measured. The δ1 and δ2 are errors associated with Y1 and Y2, respectively, which includes both errors due to unobserved confounders as well as measurement errors. (TIF) [file pone.0293510.s001.tif]
