# Supplementary material for: Approaches to estimate bidirectional causal effects using Mendelian randomization with application to body mass index and fasting glucose
Source: PLoS One. 2024 Mar 8;19(3):e0293510. doi: 10.1371/journal.pone.0293510 (PMC10923437; doi:10.1371/journal.pone.0293510)
Supplement: S1 Appendix — (PDF) [file pone.0293510.s002.pdf]

Derivation for bidirectional Mendelian randomization model

Recall, the bidirectional model is represented by joint system of equations:

$$Y_1 = \beta_{01} + \beta_{11}X_1 + \beta_{CY1}C + \gamma_{21}Y_2 + \varepsilon_1$$

$$Y_2 = \beta_{02} + \beta_{22}X_2 + \beta_{CY2}C + \gamma_{12}Y_1 + \varepsilon_2$$

In this model, the bidirectional relationship between Y_1 and Y_2 leads to a feedback loop between these two outcomes. After each feedback cycle, values of outcome variables Y_1 and Y_2 are altered. The transitional feedback steps are described in S1 Fig.

$$Y_{2.1} = \beta_{02} + \beta_{22}X_2 + \beta_{CY2}C + \gamma_{12}Y_{1.1} + \varepsilon_{2.1}$$

If we substitute the $Y_{2.1}$ in the equation $Y_{1.2}$, we get:

$$\begin{aligned} Y_{1.2} &= \beta_{01} + \beta_{11}X_1 + \beta_{CY1}C + \gamma_{21}Y_{2.1} + \varepsilon_{1.2} \\ &= \beta_{01} + \beta_{11}X_1 + \beta_{CY1}C + \varepsilon_{1.2} + \gamma_{21}(\beta_{02} + \beta_{22}X_2 + \beta_{CY2}C + \gamma_{12}Y_{1.1} + \varepsilon_{2.1}) \\ &= \beta_{01} + \beta_{11}X_1 + \beta_{CY1}C + \varepsilon_{1.2} + \gamma_{21}(\beta_{02} + \beta_{22}X_2 + \beta_{CY2}C + \varepsilon_{2.1}) + \gamma_{12}\gamma_{21}Y_{1.1} \end{aligned}$$

We now substitute the $Y_{1.2}$ in the equation $Y_{2.2}$, we get:

$$\begin{aligned} Y_{2.2} &= \beta_{02} + \beta_{22}X_2 + \beta_{CY2}C + \gamma_{12}Y_{1.2} + \varepsilon_{2.2} \\ &= \beta_{02} + \beta_{22}X_2 + \beta_{CY2}C + \varepsilon_{2.2} + \gamma_{12}(\beta_{01} + \beta_{11}X_1 + \beta_{CY1}C + \varepsilon_{1.2}) \\ &\quad + \gamma_{12}\gamma_{21}(\beta_{02} + \beta_{22}X_2 + \beta_{CY2}C + \varepsilon_{2.1}) + \gamma_{12}^2\gamma_{21}Y_{1.1} \end{aligned}$$

Resubstituting $Y_{2.2}$ in the equation again $Y_{1.3}$, we get:

$$\begin{aligned} Y_{1.3} &= \beta_{01} + \beta_{11}X_1 + \beta_{CY1}C + \gamma_{21}Y_{2.2} + \varepsilon_{1.3} \\ &= \beta_{01} + \beta_{11}X_1 + \beta_{CY1}C + \varepsilon_{1.3} + \gamma_{21}(\beta_{02} + \beta_{22}X_2 + \beta_{CY2}C + \varepsilon_{2.2}) \\ &\quad + \gamma_{12}\gamma_{21}(\beta_{01} + \beta_{11}X_1 + \beta_{CY1}C + \varepsilon_{1.2}) + \gamma_{12}\gamma_{21}^2(\beta_{02} + \beta_{22}X_2 + \beta_{CY2}C + \varepsilon_{2.1}) \\ &\quad + \gamma_{12}^2\gamma_{21}^2Y_1 \end{aligned}$$

After n substitutions, the coefficients can be represented as a geometric series as follows:

$$Y_{1.n} = (\beta_{01} + \beta_{11}X_1 + \beta_{CY1}C)(1 + \gamma_{12}\gamma_{21} + (\gamma_{12}\gamma_{21})^2 + \cdots + (\gamma_{12}\gamma_{21})^{n-1})$$
$$+ \gamma_{21}(\beta_{02} + \beta_{22}X_2 + \beta_{CY2}C)(1 + \gamma_{12}\gamma_{21} + (\gamma_{12}\gamma_{21})^2 + \cdots + (\gamma_{12}\gamma_{21})^{n-1})$$
$$+ (\gamma_{12}\gamma_{21})^n Y_1 + \varepsilon_{1.n}$$

When $|\gamma_{12}\gamma_{21}| < 1$ and n goes infinite, $(\gamma_{12}\gamma_{21})^n$ converges to 0 and Y_1 can be written as:

$$Y_1 = \frac{\beta_{01} + \gamma_{21}\beta_{02}}{1 - \gamma_{12}\gamma_{21}} + \frac{\gamma_{21}\beta_{CY2} + \beta_{CY1}}{1 - \gamma_{12}\gamma_{21}}C + \frac{\beta_{11}}{1 - \gamma_{12}\gamma_{21}}X_1 + \frac{\gamma_{21}\beta_{11}}{1 - \gamma_{12}\gamma_{21}}X_2 + \delta_1$$

Similarly, Y_2 can be written as:

$$Y_1 = \frac{\beta_{01} + \gamma_{21}\beta_{02}}{1 - \gamma_{12}\gamma_{21}} + \frac{\gamma_{21}\beta_{CY2} + \beta_{CY1}}{1 - \gamma_{12}\gamma_{21}}C + \frac{\beta_{11}}{1 - \gamma_{12}\gamma_{21}}X_1 + \frac{\gamma_{21}\beta_{11}}{1 - \gamma_{12}\gamma_{21}}X_2 + \delta_1$$

MR assumptions

Although the relationship between Y_1 and Y_2 is recursive, the instruments variables X_1 and X_2 are chosen for the observed data Y_1 and Y_2 such that MR assumption are satisfied. Particularly, we chose X_1 directly associated with Y_1 but is not associated with Y_2 conditional on Y_1. The instrumental variable X_2 was chosen similarly.
